# Psychometric properties of the Child-OIDP and oral health-related quality of life (OHRQoL) in secondary schools in Suva, Fiji

**DOI:** 10.1186/s12955-022-01953-7

**Published:** 2022-03-19

**Authors:** Anumala Ram, Masoud Mohammadnezhad, Tamara Mangum, Brian Mangum

**Affiliations:** 1 Department of Oral Rehabilitation, School of Dentistry and Oral Health, College of Medicine, Nursing and Health Sciences, Fiji National University, Suva, Fiji; 2 School of Public Health and Primary Care, Fiji National University, Suva, Fiji; 3 Antigua School of Medicine, University of Health Sciences, San Juan, Puerto Rico

**Keywords:** Oral health-related quality of life, Daily performance, School children, Dental caries, Periodontal disease, Fiji

## Abstract

**Background:**

Oral health-related problems are highly prevalent and, like many other diseases, affect Quality of Life. Although most primary schools in Fiji have supervised school brushing programs and have regular screenings these preventive aspects are missing in secondary schools.

**Objective:**

To assess the internal consistency reliability, face and content validity of the Child-OIDP questionnaire and determine the oral health-related quality of life in 15-year-olds in Suva, Fiji.

**Methods:**

A cross-sectional prospective study was carried out on 15-year-old children from four secondary schools in Suva, Fiji from 2014 to 2015. All students enrolled in the 10th and 11th year of studies were included. Multi-stage cluster sampling was used to identify the participants and the sample size of 367 was calculated. The Child Oral Impact on Daily Performance (Child-OIDP) self-administered questionnaire was used to collect data and data was analyzed using Epi-Info (3.5.1).

**Results:**

A total of 281 students (76.6%) responded, of whom 47.0% experienced at least one impact. Cronbach’s alpha for the Child-OIDP frequency items was 0.70 and the corrected item-total correlation ranged from 0.13 to 0.57. The most common performances that were affected were eating (27.8%) and relaxing (12.8%). Performances that were *severely* and *most severely* influenced were social contact (23.1%), smiling (16.7%) and relaxing (16.7%). The most common conditions leading to impacts were dental sensitivity (38.4%), dental caries (23.5%) and toothache (21.4%).

**Conclusion:**

The original version of the Child-OIDP is a reliable index with acceptable internal consistency when used directly in the Fiji setting, however, further studies to validate the tool will be useful. Oral impacts were prevalent, but not severe.

## Introduction

Oral diseases encompass chronic conditions that affect teeth and other oral structures. Common oral diseases include dental caries, periodontal disease, and malocclusion. Even though oral diseases are preventable, more than 3.5 billion people around the globe are affected [1]. Dental caries is the most common oral condition and is more prevalent in low to middle-income countries and affects just over half of the permanent teeth in children [2].

Oral health-related problems are prevalent and, like many other diseases, affect various aspects of life, including social, economic, physical, and psychological aspects [3]. The association between clinical dental status and quality of life (QoL) is well established. Studies in a number of countries have reported compromised QoL in school-age populations due to dental caries [4–13]. Oral diseases can lead to pain, infection, diminished QoL, missed school days and reduced concentration/ productivity, family disruptions and financial burdens [1]. It is essential to evaluate individuals’ opinions of their health and wellbeing together with the existence or absence of disease to precisely develop health promotion strategies, establish programs for disease prevention and assign resources for health care. Allen (2003) describes that patient’s evaluation of how their health affects their QoL is usually very different from the heath care professionals’ assessment [3]. For this reason, it is essential that patients’ own evaluations is also considered when delivering health care.

QoL has been defined by the World Health Organization (WHO) as “individuals’ perception of their position in life in the context of the culture and value systems in which they live and in relation to their goals, expectations and concerns” [14]. According to Allen (2003), Oral Health-Related Quality of Life (OHRQoL) indices were created because objective clinical measures of diseases provided insufficient insight into the effects of oral diseases and their effects on daily living and QoL [3]. OHRQoL is made up of autonomous components such as clinical indicators of oral health, assessment of general and oral health and physical, social and psychological functioning, but at the same time these components are connected. Quality of life has been established as an important determinant of care seeking, adherence to treatment regimen, satisfaction with the care received, and as an outcome for evaluating the impact of treatments [11]. Some of the advantages of assessing OHRQoL include assisting clinical practitioners in deciding on different treatment modalities and evaluating their outcomes, assisting researchers in recognizing health determinants, measuring levels of risk factors for health and monitoring the utilization of health care services. It also assists policymakers in developing programs and determining precedence, strategies and funding decisions at different levels [11].

Fiji is comprised of more 300 islands located in the Melanesian zone of the Pacific, with approximately 90% of the population residing in two main islands, Viti Levu and Vanua Levu [15]. According to the Fiji National Oral Health Survey (NOHS) 2004, the overall prevalence of untreated dental caries (tooth decay) in permanent teeth was 55.7%, with 14.6% having four or more decayed permanent teeth. Approximately 80% of the participants had signs of caries involving their permanent teeth (including decay, missing and filled teeth); among which 18.9% of 6-year-olds, 52.3% of 12-year-olds, 67.5% of 15–19-year-olds, 98.1% of 35–44 year-olds, and 99.5% of 55–64 year-olds were affected. A similar pattern was seen with periodontal disease (gum disease), where the severity of disease increased substantially with increasing age across all age groups. Data on the periodontal status of the worst sextant revealed that 67.1% of the participants had calculus, 3.3% had bleeding, 19% had shallow pockets, and 4.1% had deep pockets [16].

As in other countries, Fijian children are at risk of dental diseases. Although most primary schools in Fiji have supervised school brushing programs and have regular screenings by the school dental teams, these preventive aspects are missing in secondary schools. This group also marks the transition where physical appearance and self-esteem have high importance, and this also relates to the appearance of teeth [17]. Assessing OHRQoL among 15-year-olds is important because by than all of the permanent teeth (excluding the wisdom teeth/third molars) have erupted. There is scarce data on the oral health status of adolescents, and so this study will also provide important information on the oral health and OHRQoL of this group.

There are no data on the impact of oral diseases on QoL in Fiji. Hence, this study is an essential step towards assessing and evaluating the effects of oral conditions on QoL in 15-year-olds in Suva. The study aimed to assess the internal consistency reliability, face and content validity of the Child-OIDP questionnaire and determine the oral health-related quality of life in 15-year-olds in Suva, Fiji.

## Methodology

### Study design and setting

A cross-sectional study was carried out on 15-year-old school children in Suva, Fiji, in 2014–2015. Suva as capital city of Fiji includes about 57 per cent of the students enrolled in schools in Fiji. Four high schools (Saint Joseph’s Secondary School, Jai Narayan College, Dayanand and Anglo Vedic College and Suva Sangam High School) were randomly selected to conduct this study.

### Study population and sample

Data were obtained from the Fiji Ministry of Education for students enrolled in the 10^th^ and 11^th^ year of studies as these students would fall in the 15-year-old age group. Students without parental or guardian consent or those who were not willing to participate in the study and those with medical conditions that contraindicate oral assessments were excluded.

Multi-stage cluster sampling was used to identify the participants. Data from the Ministry of Education to divide the city into geographical wards. The city Suva is made up to six wards, out which three wards were randomly selected. From each of these three wards, four schools were randomly selected. In Fiji, there were 27,550 students enrolled in year 10 and 11, with 57% (15,703 potential participants) of those located in Suva. Using a RaoSoft Digital Sample Size Calculator (RaoSoft, Inc., Seattle, WA) and considering a confidence level of 95%, margin of error of 5%, and respond distribution of 65%, a sample size of 274 was chosen for this study. All the eligible participants’ names were placed in a jar and picked using a lottery method to minimize bias.

### Oral assessment form

Oral assessments were done to evaluate the dental status as the first part of the study. The Decayed, Missing and Filled Teeth (DMFT) and Community Periodontal Index (CPI) indices were used [18]. Caries status was assessed for all teeth, and periodontal assessments involved index teeth only. The clinical examination was carried out using the World Health Organization (WHO) protocol and criteria (one single examiner, in natural light, after drying and isolating teeth); using a WHO probe and flat mirror. The oral assessment was carried out by a trained dentist and standardized protocols were followed.

### Child oral impact on daily performance (Child-OIDP) questionnaire

The Child-OIDP [13, 19] has two parts and was self-administered. In Fiji the school curriculum is delivered in English and the Child-OIDP questionnaire has been previously validated in English [19]. However, to assess the validity of the questionnaire it was tested among few randomly selected students to assess if the questionnaire was understood by the participants. Minor changes were done to the questionnaire and applied in the main study. The first part of the questionnaire consisted of a check-box style list of oral pathological conditions that affected the participants during the past three months. The goal of the first part of the questionnaire was to familiarize the participants to oral diseases/conditions that would have affected their QoL.

The second part of the Child-OIDP questionnaire was used to assess the effects of oral conditions on the eight performances (1) eating, (2) speech, (3) oral hygiene, (4) sleeping and resting, (5) feeling emotional, (6) smiling, the ability to show one’s teeth without embarrassment, (7) study, lessons, learning, going to school and (8) socializing. The reference period was 3 months. The “frequency” and “severity” of the effects of oral conditions that the participants recognized as affecting their everyday performances was determined by summing the responses to the statements with the “frequency” (0–3) and “severity” (0–3) scores. The “perceived intrusion” was evaluated, as the reasons that the participants recognize to cause oral impacts, the questions were supplemented with a list of indicators in the second step. The score for the perceived oral effects for everyday performances was calculated by multiplying the “frequency” and “severity” scores. The final score for one performance (impact intensity) ranged from zero to nine. The mean (the prevalence) of the impacts of oral health on everyday performances of the participants was calculated as a percentage of the maximum obtainable scores. Cronbach’s alpha was calculated to determine the psychometric property of the tool. Since the school system in Fiji is in English, all the questionnaires were administered in English.

The questionnaire was piloted on eight child participants at the JB Savou Teaching Dental Clinic in Suva, Fiji. Minor amendments were then done to the existing questionnaire based on the pre-test.

### Study procedure

Once approval was granted from the school heads, consent letters, participant information sheets and copies of approvals from the Ministry of Education and the Fiji National Health Research Ethics Committee (FNHREC) were given to 91 randomly selected one week prior to the data collection. Data were collected only from participants who presented their approved consent forms signed by their legal guardians. Final consent was obtained from the participants using assent forms.

Clinical examinations were carried out with the participants sitting on a chair or bench (whichever was available at the schools) and the examiner standing behind the participant. All clinical examinations were carried out by a single qualified dentist and data was recorded by a single administrative assistant. The Child-OIDP questionnaire was completed independently by the participants; however, the investigator provided some assistance, such as how to fill in the questionnaires and explaining the options.

### Data management and analysis

The data collected were coded, collated, and cleaned manually first, then entered into Epi-Info (3.5.1) for analysis. Spearman’s correlation was used to determine inter-item consistency of Child-OIDP items. Following the computation of Child-OIDP scale scores, the prevalence of one or more impacts was determined as a percentage of the maximum obtainable score. Cross-tabulations were used to examine the magnitude of differences in impact prevalence by oral conditions, along with their statistical significance (*P* < 0.05).

### Ethical considerations

Prior to data collection, ethical approvals were obtained from the College Health Research Ethics Committee (CHREC) at the Fiji National University, the Fiji National Health Research Ethics and Review Committee (FNHRECRC) and the Ministry of Education. Approvals were also obtained from heads the selected schools. All participants’ parent or guardian were asked to sign an informed assent form before collecting data.

## Results

A total of 281 15-year-olds participated in this study, comprising 182 (64.4%) females and 100 (35.6%) males, with a response rate of 77.8%. Some 132 (47%) were *Itaukei* (indigenous Fijians), 105 (37.4%) Fijians of Indian Origin, and 44 (15.7%) Fijians of Other Origin (Table 1).

**Table 1:** Characteristics of participants

Characteristics	n	%
Gender		
Females	181	64.4
Males	100	35.6
Ethnicity		
*I-taukei* (indigenous Fijians)	132	46.96
Fijians of Indian Origin	105	37.37
Fijians of Other Origin	44	15.66

### Psychometric properties of the Child-OIDP

Since the Child-OIDP performance scores were non-parametric, Spearman’s correlation coefficient scores were used to determine the inter-item correlation coefficients (Table 2). Those ranged from 0.08 (relationship Speech and Relaxing) to 0.65 (Smiling and Social Contact).

**Table 2:** Spearman’s correlation between single items of the Child-OIDP performance score

	Eating	Speech	Cleaning	Relaxing	Emotion	Smiling	Studying	Social contact
Eating	1							
Speech	− 0.05	1						
Cleaning	0.27	− 0.04	1					
Relaxing	0.43	0.08	0.18	1				
Emotion	0.09	0.13	0.10	0.44	1			
Smiling	0.10	0.16	0.10	0.19	0.24	1		
Studying	0.22	0.15	0.11	0.51	0.45	0.35	1	
Social contact	0.09	0.12	0.21	0.38	0.28	0.65	0.48	1

Cronbach’s alpha score for all the items for the total sample was 0.70; the corrected item-total for the Child-OIDP correlation ranged from 0.130 to 0.576 (Table 3). Face and content validity were tested during the pilot study on eight 15-year-olds. The validation process included assessment of the questionnaire in the Fijian context in terms of culture, sensitivity of the questionnaire and content. Two public health experts were involved in this process. Minor amendments were then done to the existing questionnaire based on the pre-test, which included spelling errors and other grammatical corrections. No changes to the actual content of the original Child-OIDP tool were made.

**Table 3:** Reliability analysis: corrected item-total correlations

	Corrected item-total correlation	Cronbach’s alpha if item deleted
Eating	0.287	0.706
Speech	0.130	0.715
Cleaning	0.238	0.698
Relaxing	0.576	0.618
Emotion	0.415	0.663
Smiling	0.413	0.663
Study	0.576	0.636
Contact	0.562	0.635

Standardized Cronbach’s alpha 0.698–0.70

### Prevalence, characteristics, and severity of oral impacts

Table 4 illustrates the mean scores for Child-OIDP among the participants for individual performances. The mean scores of impacts on each of the eight performances ranged from 0.16 (± 0.94) to 0.61 (± 1.47) (maximum possible score is 9). Mean impact scores for eating (0.61 ± 1.47) and relaxing (0.34 ± 1.26) were the highest while those for study (0.17 ± 0.88) and social contact (0.16 ± 0.94) were the lowest. Overall, 132 (46.98%) participants reported at least one or more impacts on daily performance and ranged from 1 to 8 “performances with impacts” (PWI).

**Table 4:** Prevalence, score and intensity of oral impacts

	Overall	Eating	Speech	Cleaning	Relaxing	Emotion	Smiling	Studying	Social contact
Prevalence									
N = 281	n = 132	n = 78	n = 26	n = 32	n = 36	n = 20	n = 29	n = 18	n = 13
Prevalence %	47.0	27.8	9.3	11.4	12.8	7.1	10.3	6.4	4.6
Impact score									
Range	0–72	0–9	0–9	0–9	0–9	0–9	0–9	0–9	0–9
Mean (SD)	2.2 (5.5)	0.6 (1.5)	0.2 (0.9)	0.3 (1.0)	0.3 (1.3)	0.2 (1.1)	0.3 (1.1)	0.2 (0.9)	0.2 (0.9)
Impact intensity (% with impact)									
Very little	56	62.8	53.9	50.0	61.1	40.0	58.7	55.6	38.5
Little	12.7	12.8	23.1	25.0	5.6	5.0	6.9	5.6	15.4
Moderate	17.9	12.8	11.5	15.6	16.7	40.0	20.7	22.2	23.0
Severe	7.1	7.7	7.69	6.3	8.3	5.0	3.5	11.1	7.7
Very severe	6.4	3.9	3.9	3.1	8.3	10.0	10.4	5.6	15.4

The most common performances that were affected in this study were eating (27.8%) and relaxing (12.8%). The least common performances that were affected were social contact (4.6%) and study (6.4%). Among the participants, 7.1% and 6.4% reported severe and very severe intensity of impacts, respectively. The intensity of impacts for all activities revealed that social contact (23.1%), smiling (16.7%) and relaxing (16.7%) were severely and most severely influenced. Eating, speech and tooth cleaning had the lowest levels of impact intensity.

### Causes of oral impacts

The causes of oral impacts on specific activities are shown in Table 5. The most common conditions leading to impacts were dental sensitivity (38.4%), dental caries (23.5%) and toothache (21.4%). Additionally, oral conditions associated with appearance of teeth and gingival problems like modified (unpleasant) color of teeth (12.5%), swollen gums (11.8%) and bad breath (10.8%) commonly affected the participants.

**Table 5:** Frequency of oral conditions perceived as causing overall oral impacts

Oral conditions causing overall impacts	Frequency n (%)
Tooth ache	60 (21.4)
Dental sensitivity	108 (38.4)
Dental caries (decay)	66 (23.5)
Fractured tooth	29 (10.3)
Modified (unpleasant) color of teeth	35 (12.5)
Shape or number of teeth	19 (6.8)
Position of teeth	23 (8.2)
Bleeding gums	44 (15.7)
Swollen gums	33 (11.8)
Plaque	7 (2.5)
Bad breath	30 (10.8)
Painful ulcerations (sores) inside the mouth	13 (4.6)
Moving of deciduous (milk) teeth	1 (0.4)
Growing (erupting) permanent teeth	8 (2.9)
Missing teeth	15 (5.3)
Mouth or face deformities (cleft palate or lips)	1 (0.4)
Extracted permanent tooth (teeth)	9 (3.2)

The toothache and dental sensitivity were most common perceived causes of impacts that affected the daily activities that impacted eight and five performances, respectively (Table 6). The common impacts on eating were due to toothache (16.7%) and dental caries (6.0%). Dental caries was the most common perceived cause of impacts on six everyday activities: study, emotion, sleeping, tooth cleaning, speech, and eating. Shape and number of teeth and modified color of teeth were the most common perceived cause of impacts on smiling.

**Table 6:** Main oral conditions causing impacts on each of the eight performances

	Study	Smiling	Emotion	Relaxing	Tooth Cleaning	Speech	Eating	Social Contact
	n (%)							
Toothache	13 (72)	–	9 (45)	23 (61)	8 (24)	8 (37)	47 (56)	3 (22)
Dental caries	2 (11)	5 (16)	5 (25)	7 (18)	4 (13)	3 (10)	11 (13)	–
Dental sensitivity	3 (17)	–	6 (30)	4 (10.5)	10 (30)	–	19 (23)	–
Shape and number of teeth	–	7 (23)	–	–	–	–	–	2 (14)
Swollen gums	–	–	–	4 (10.5)	11 (33)	–	7 (8)	–
Modified colour of teeth	–	9 (29)	–	–	–	5 (17)	–	–
Position of teeth	–	6 (19)	–	–	–	7 (23)	–	–
Bleeding gums	–	–	–	–	–	4 (13)	–	–
Fractured teeth	–	4 (13)	–	–	–	–	–	–
Bad breath	–	–	–	–	–	–	–	9 (64)

## Discussion

### Prevalence, characteristics, and severity of oral impacts

The prevalence of oral impacts experienced by the participants of the study during the past three months was 47.0%. That is, 132 out of 281 participants reported at least one or more impacts. The prevalence reported in the current study is lower than the other studies on similar age groups that used the same tool (Child-OIDP). The Thai study [13] showed that the prevalence of oral impacts was 89.8%; the Brazilian study [20] showed that the prevalence was 80.7%; the French study [21] showed that the prevalence was 73%; the Malay study [22] showed that prevalence was 66.7%; the Ugandan study [23] showed that the prevalence was 62% and the Sudanese study [6] showed that the overall prevalence was 54.6%. Differences in the severity of disease, cultural variations, age differences, and location of the participants may partly explain the reasons for the differences in the overall impact in different studies. In the present study, face validity was conducted even though the Child-OIDP is a standard validated questionnaire. This is because Fiji is ethically very diverse and it was essential to determine if the tool was understood by the participants.

There were other studies where the prevalence of the overall oral impacts were lower than in the current study: 8.6% in the Tanzanian study [24], 30.0% in the Spanish study [25], and 40.4% in the English study [19]. This implies that either the prevalence of oral conditions/diseases is low in these studies or the participants’ QoL is not affected by the burden of oral diseases or both.

The mean Child-OIDP score for the current study was 2.2 ± 5.0, which is more than those reported in other studies. For instance, in the Sudan study [6] the Child-OIDP score was 1.4 ± 1.7 and in the Italy study [26] the score was 1.9 ± 3.7. However, the mean Child-OIDP in the current study was much lower in comparison to most studies that used the same tool and looked at similar age groups. One of the reasons for the low Child-OIDP values in the current study may be because the participants’ may not perceive that the oral conditions/diseases have an impact on their QoL. For instance, in the Thailand study [4] the Child-OIDP score was 7.8 ± 7.8, in the France study [21] the score was 6.3 ± 8.2, and in the Brazil study [20] the score was 9.2 ± 10.1.

Among the participants with impacts, the range of impacts varied from one to eight PWI. This result is similar to the findings of other studies done in other settings using the same tools, such as in Sudan [6], Thailand [13], England [19], Brazil [20] and Malaysia [22]. In the current study, 54.5% of the participants reported at least one PWI, 17.4% had two PWI, 17.4% had three PWI, and 6.1% had four PWI. Few participants had five or more PWIs, 4.6%.

Even though the overall prevalence of impacts in the current study was lower than most studies mentioned above, the individual performances with impacts was higher in the current study. In the Thai study [13], about 16.2% of the participants had at least one PWI, in the Sudan study [6] it was 18.1%, and in the England study [19] it was 40.4%. On the other hand, there were some studies where the individual performances with impacts were higher than in the current study, such as the Malaysia study [22] where it was 66.7% and Brazil [20] study where it was 80.7%. In the current study few participants had severe or very severe intensity of impacts the findings were very similar to the findings from the Thai study [13]^.^

Despite the findings of the study showing that oral impacts are prevalent in this current study population, they were not reported to be severe. Most of the clinical causes that added to the prevalent impacts are self-limiting, for instance oral ulcers. The current study reported that eating was the most significant aspect of oral health-related quality of life of the participants.

In relation to the most prevalent oral impacts, eating and relaxing were the two performances that were mostly affected in the current study; however, the intensity of the impact was not severe. The performances on which the intensity of impact was most severe were study and contact, and these two performances were the least prevalent. These three performances, eating, speech and cleaning had low levels of severe and very severe intensity of impacts. The findings of the current study were similar to those reported in other studies. Eating was the most prevalent performance that had the highest reported impacts in other studies [6, 13, 19–22, 24–27] as well. Other prevalent performances with impacts were emotional status in certain studies [13, 20], and cleaning, as shown in other studies [6, 19, 22, 24–27].

### Causes of oral impacts

The participants perceived several oral and dental problems as the causes that had impacts on their general oral health. The problems that were reported to be most prevalent and led to oral impacts were dental sensitivity, dental caries, toothache and bleeding gums. This finding was consistent with other studies [13, 20, 25–27]. Other studies reported of other perceived dental problems as more prevalent, such as badly positioned teeth [21]; toothache [24, 27]; and erupting teeth [6].

Additionally, oral conditions associated to appearance and gums frequently affected the participants, modified (unpleasant) color of teeth, swollen gums and bad breath (10.8%) were quite frequently cited. These findings were not reported in many studies; however, they were consistent with two of the other studies [21, 27].

### Limitations

Although this is the first study conducted in Fiji there were a few limitations such as one of the schools that were selected was an all-girls school and this tipped the balance of gender in the study towards female participants. Due to this limitation gender-based analysis and reporting was not done in this study. The data from the questionnaire is self-reported. As much as self-reported data provides useful information, there is always possibilities of selective memory (participants may or may not recall the events that occurred have few months ago); telescoping (recalling events that occurred at one time as if they occurred at another time), in the study the period of interest was 3 months; possible bias might have been incorporated if the participants recalled events that would have occurred more than three months ago. Attributions may have led to over or underreporting of both negative and positive aspects. Exaggeration (the act of representing outcomes or events as more significant than in reality), this could have led to reporting disease as more severe than it was. The inclusion of questions related to socio-economic status in the questionnaire would have given more scope for analysis and comparison of oral health-related quality of life to social indicators. The Spearman’s correlation between some domains were low; the possible reasons for this could be variability in data, lack of linearity, presence of outliers, errors in measurement and characteristics of the sample. This is expected when samples are selected from a diverse population pool. A post-hoc analysis was not done in this study, however, this would be provided useful insights and is recommended for future research. Even though the original version of the Child-OIDP has shown acceptable internal consistency when used directly in the Fiji setting, concurrent validity tests would be useful to validate this index and is recommended for future studies. It is also recommended that the test–retest reliability of the Child-OIDP questionnaire is carried out in future studies to fully evaluate psychometric properties of the tool in the Fijian context.

The general implications of this research are that it was able to fill the gap in knowledge and provide baseline information regarding OHRQoL in adolescents in Suva, Fiji. It was also able to provide a clear picture of the burden of the two most common oral diseases, dental caries and periodontal disease, in 15-year-olds in Suva, Fiji. With this information at hand, it was easier to answer the question regarding the effects of oral diseases/conditions on QoL.

## Conclusion

This study showed that the original version of the Child-OIDP is a reliable index with acceptable internal consistency when used directly in the Fiji setting, however, further studies to validate the tool will be useful. Oral health has an effect on quality of life in 15-year-olds in Suva. Oral impacts reported by the participants were prevalent, but not severe. The most common performances that were affected by oral conditions were eating and relaxing. The more prevalent problems leading to impacts were dental sensitivity, dental caries, toothache, and bleeding gums.

## Data Availability

Data cannot be shared publicly due to participant confidentiality and approval from participant was not obtained for public sharing. Deidentified data can be made available to those researchers who meet the criteria for access to confidential data.

## Abbreviations

OHRQoL: Oral health-related quality of life
OIDP: Oral impact on daily performances
PWI: Performances with impact
QoL: Quality of life
